# Identifying Barriers Faced by Applicants without a Home Residency Program when Matching into Plastic Surgery

**DOI:** 10.1055/a-2202-9219

**Published:** 2024-02-07

**Authors:** Steven L. Zeng, Gloria X. Zhang, Denisse F. Porras, Caitrin M. Curtis, Adam D. Glener, J. Andres Hernandez, William M. Tian, Emmanuel O. Emovon, Brett T. Phillips

**Affiliations:** 1 Duke University School of Medicine, Durham, North Carolina; 2 Department of Plastic and Reconstructive Surgery, Wake Forest University, Winston Salem, North Carolina; 3 Department of Plastic and Reconstructive Surgery, Duke University, Durham, North Carolina; 4 Department of Plastic Surgery, Duke University School of Medicine, Durham, North Carolina

**Keywords:** plastic surgery, residency match, diversity

## Abstract

**Background** Applying into plastic surgery (PS) is competitive. Lacking a home residency program (HRP) is another barrier. Our goal is to characterize challenges faced by PS applicants without HRPs and identify solutions.

**Methods** Surveys were designed for current integrated PS residents and applicants in the 2022 Match without HRPs. Surveys were distributed electronically. Only U.S. allopathic graduate responses were included.

**Results** Of 182 individuals surveyed, 74 responded (39%, 33 residents, 41 applicants). Sixty-six percent reported feeling disadvantaged due to lack of an HRP. Seventy-six percent of applicants successfully matched. Of these, 48% felt they required academic time off (research year) versus 10% of unmatched applicants. Ninety-seven percent of matched applicants identified a mentor versus 40% of unmatched applicants ( *p* < 0.05). Matched applicants identified mentors through research (29%) and cold calling/emailing (25%). Matched versus unmatched applicants utilized the following resources: senior students (74 vs. 10%, *p* < 0.05) and social media (52 vs. 10%, *p* < 0.05). Among residents, 16 had PS divisions (48%). Thirty-six percent with divisions felt they had opportunities to explore PS, compared with 12% without divisions. Residents without divisions felt disadvantaged in finding research (94 vs. 65%, *p* < 0.05), delayed in deciding on PS (50 vs. 28%), and obtaining mentors (44 vs. 35%) and letters of recommendation (31 vs. 24%).

**Conclusion** PS residents and applicants without HRPs reported feeling disadvantaged when matching. The data suggest that access to departments or divisions assists in matching. We identified that external outreach and research were successful strategies to obtain mentorship. To increase awareness for unaffiliated applicants, we should increase networking opportunities during local, regional, and national meetings.

## Introduction

In the United States, plastic surgery (PS) is highly competitive, with a total of 419 applicants and 194 positions in 2022. 1 2 Furthermore, the number of total positions only increased by four between 2021 and 2022, growing the discrepancy between the number of applicants and positions. 3 Competition will likely continue to grow, impacting both students pursuing PS and program directors evaluating applicants.

Due to the growing number of applicants who fail to match into PS, there has been increased interest in identifying factors that influence success as well as barriers that may disadvantage applicants. 4 5 Past research shows that several factors such as test scores, research productivity, letters of recommendation (LOR), and performance on away rotations are highly important when evaluating candidates as well as if a candidate has previously failed to match. 6 7 8 A recent study also found that graduates from allopathic medical schools without an affiliated integrated residency program comprised 24.4% of successfully matched applicants, while those with affiliated programs comprised 72.2%. Additionally, at the top quartile residency programs, applicants without a home residency program (HRP) comprised only 17.4% of residents. 9 10

A study aimed at measuring the concerns of students without an HRP found that almost half consider themselves to be underrepresented and that their initial exposure to PS came through shadowing. Only 10% reported being exposed to PS through their school's curriculum and more than half mentioned they did not have any professional exposure to PS. Most students reported an inability to identify a mentor, and all reported difficulties securing subinternships. Importantly, more than half of students reported being extremely concerned about matching. 11 12 13 14

Additionally, with the implementation of a P/F United State Medical Licensing Examination (USMLE) Step 1 score, students without an HRP might be at an even greater disadvantage in the Match process. 15 16 17 The USMLE Step 1 test has been historically used as an objective measure to distinguish students. With fewer objective measures moving forward, school names, professional networking, and LOR will likely hold greater value. 18 Furthermore, programs might also have trouble evaluating applicants successfully, which may be compounded by an increase in students applying to competitive surgical specialties. 18 Ultimately, these changes could generate potential challenges, associated with an emphasis on more subjective measures. Additionally, the coronavirus disease 2019 pandemic led to restrictions on away rotations, and students without an HRP had fewer opportunities to gain experience and mentorship. 19 20 21 While restrictions have eased, it is unclear whether opportunities for away rotations will return to the prepandemic years.

There is paucity in the literature examining the direct impact of medical students without an HRP. The goal of the present study is to investigate whether having an HRP confers an advantage to applicants. Additionally, the authors hope to identify challenges that applicants without HRPs face and propose solutions that aim to address disparities in the Match.

## Methods

Two separate surveys were designed for integrated PS applicants from the 2022 match and residents who attended a medical school without an HRP. The surveys were distributed via Qualtrics and were emailed three times over the course of 3 weeks to encourage response. In the applicant survey, question 1 addressed their ethnicity. Questions 2 to 5 characterized their home institution, including the presence of plastic surgeons and a PS division. Questions 9 and 10 characterized their match process, which included whether they matched successfully to PS, the location of their residency on their rank list, involvement in a subinternship, and the number of programs to which they applied. Questions 11 to 20 addressed the applicant's perception of how their home institution impacted variables such as exposure to and the decision to pursue PS, research opportunities, mentorship, and resources used. When addressing resources used, we defined cold calling/emailing as phone or email contact without a prior relationship. Question 21 addressed the impact of COVID-19 on their application. The resident survey matched the applicant survey; however, there were additional questions that addressed any changes in their home institution since they graduated.

Applicant surveys were distributed via email to medical students who applied to our institution's integrated PS residency program in 2022 who did not have HRPs. In addition, we utilized a community-sourced Google document that contains a public list of matched 2022 applicants and included all additional medical students from schools with no HRP.

Resident surveys were distributed via email to post-graduate year 1-5s (PGY1-5s). In addition to the applicant cohort, which represents the incoming PGY-1s, our survey participants cover the current 2022 to 2023 entire resident cohort (PGY1–6). We utilized publicly available information on current residents without HRPs' addresses as well as phone numbers. For residents without publicly available contact info, we sent direct a message through available channels such as LinkedIn, Instagram, or Twitter.

We compiled a list of all integrated and independent PS programs through Accreditation Council for Graduate Medical Education (ACGME) and cross-referenced this to all the residents and medical students who received the survey. We excluded DO and internationally trained medical students/residents as well as all PGY6 residents. DO and international medical graduate (IMG) applicants were excluded as they do not have home institutions domestically and traditionally match at different rates than graduates from U.S. MD programs. PGY-6 residents were excluded out of concern for low response rate.

Statistical analysis was performed using Microsoft Excel. Categorical variables were analyzed using chi-square analysis. Independent Student's *t* -test was utilized for analyzing the difference between means of continuous variables. The threshold for statistical significance was set at *p* < 0.05. The study received Institutional Review Board (IRB) exemption from our institution's review board.

## Results

**Fig. 1:**
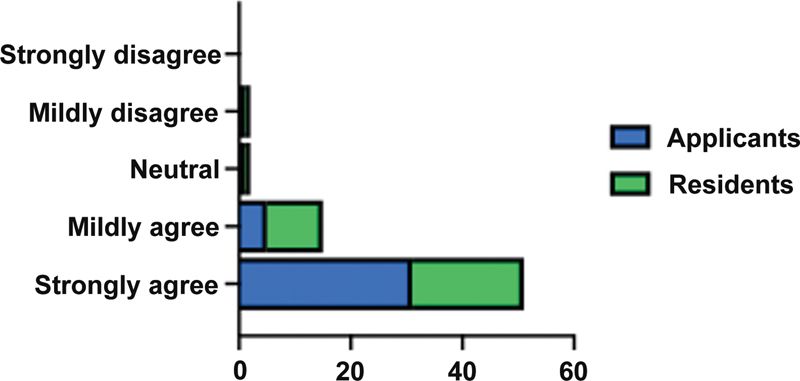
Total responses from applicants and residents (PGY1–5s) to the following question: “Do you feel that as a medical student without a home plastic surgery department that you were at a significant disadvantage matching in a plastic surgery program?”

Of the 82 applicants and 100 residents surveyed, 74 (39%) responded (33 residents, 41 applicants), resulting in a response rate of 33% for residents and 50% for applicants. Among applicants, 76% ( *n* = 31) strongly agreed that they were at a disadvantage when matching in PS ( Fig. 1 ). Sixty-one percent of residents ( *n* = 20) strongly agree that they were at a disadvantage when matching in PS ( Fig. 1 ). Collectively, roughly two-thirds of all respondents reported feeling disadvantaged in the Match due to the lack of an HRP.

The two most utilized resources by applicants were outside attendings (84%) and senior students at their institution (54%). Similarly, 85% of residents utilized outside attendings, and 62% relied on senior students. The least utilized resource among applicants was medical school advisors (19%, *n* = 7). Among residents, the least utilized resource was participation in professional societies (22%, *n* = 7).

**Fig. 2:**
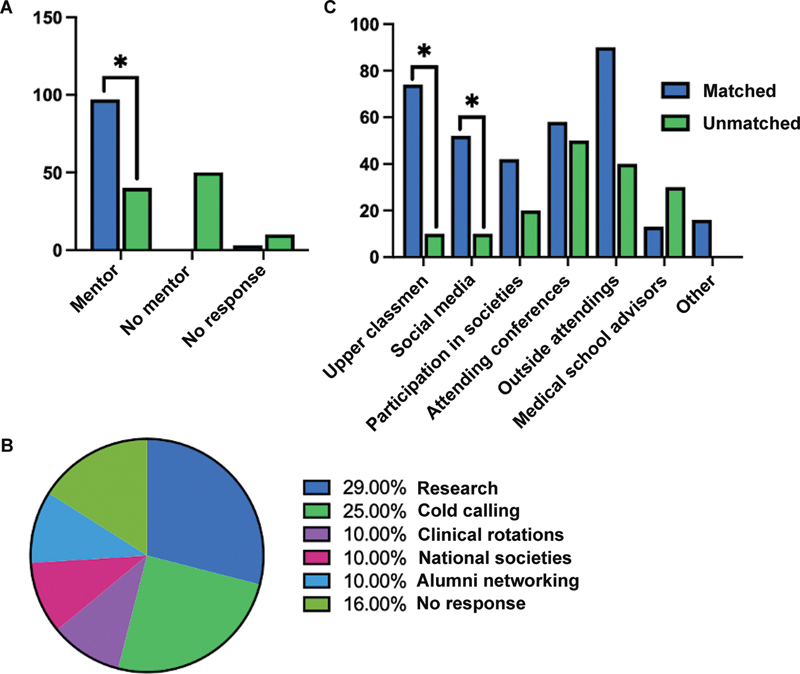
( **A** ) (Upper left) Percent of matched (97%) versus unmatched (40%) applicants showing if they had obtained a plastic surgery mentor ( *p* < 0.05). ( **B** ) (Bottom) Percent responses of resources used by matched applicants to identify a plastic surgery mentor. ( **C** ) (Upper right) Percent responses from matched versus unmatched applicants showing resources used while applying to residency. * indicates *p* < 0.05.

Among surveyed applicants without HRPs, the overall match rate was 76% in 2022. Of these, 48% strongly felt required to take an additional year to pursue research opportunities or additional clinical experiences, compared with 10% of unmatched students. We also found that students who identified a mentor were more likely to match with 97% of matched students identifying a PS mentor compared with 40% of unmatched participants ( *p* < 0.05; Fig. 2A ). The most utilized strategies to identify mentors by matched students were conducting research (29%) and cold calling/emailing (25%; Fig. 2B ). Furthermore, there were differences in usage of the following resources by matched versus unmatched students: senior students (74 vs. 10%, *p* < 0.05) and social media (52 vs. 10%, *p* < 0.05; Fig. 2C ).

**Fig. 3:**
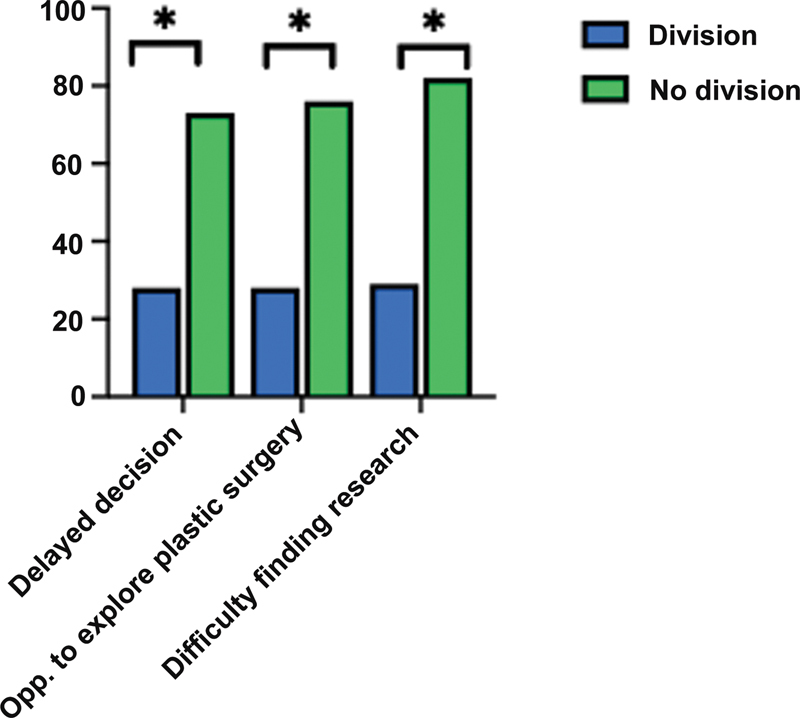
Percent responses from applicants with and without plastic surgery divisions, demonstrating their feeling delayed in their decision (28 vs. 73%, *p* = 0.022), not having opportunities to explore the field (28 vs. 76%, *p* = 0.013), and difficulty in finding research opportunities (29 vs. 82%, *p* = 0.003). * indicates *p* < 0.05.

Among surveyed applicants, 17% ( *n* = 7) had a PS division at their home institution, while 83% ( *n* = 24) did not. Of note, a PS division was designated to be distinct from having a residency program. We noted no differences in match rate (71 vs. 76%). However, we found that medical students without an HRP who also lacked a PS division perceived that they were further disadvantaged. This included feeling delayed in their decision (28 vs. 73%, *p* < 0.05), not having opportunities to explore the field (28 vs. 76%, *p* = 0.013), and difficulty in finding research opportunities (29 vs. 82%, *p* = 0.003; Fig. 3 ).

Among residents, 16 (48%) had a formal PS division. Comparing survey responses between residents with and without formal PS divisions, 36% with home divisions felt they had opportunities to explore PS, compared with 12% of those without a division. Additionally, residents without home divisions felt more disadvantaged in finding research (94 vs. 65%, *p* < 0.05), delay in deciding on PS (50 vs. 28%), and obtaining mentors (44 vs. 35%) and LOR (31 vs. 24%). Otherwise, residents with home divisions felt more limited in networking (29 vs. 19%) and obtaining subinternships (29 vs. 13%) than residents without home divisions. Residents with home divisions employed the following strategies to find PS-related research projects: contacting outside departments (35 vs. 38%), personal mentors (35 vs. 12%), contacting community physicians (11 vs. 6%), and utilizing a research year (6 vs. 6%).

## Discussion

Within the applicant pool, those without an HRP account for the minority of PS residents (26%) despite the majority of U.S. allopathic medical schools not having a PS HRP (54%). 22 Additionally, applicants without an HRP represent a disproportionately small minority at top-quartile PS residency programs. 9 However, our survey data show that those without an HRP seem to match at higher rates than the national average (76 vs. 67%). Despite many studies, including our own, demonstrating sentiments of being disadvantaged among unaffiliated applicants, few studies to date directly compare match rates between these populations. 10 While additional studies are warranted to further characterize factors influencing match rates for unaffiliated students, not having an HRP undeniably reduces an applicant's exposure to PS, their ease of access to mentorship, and their ability to conduct PS research. The PS community has already made improvements to become more inclusive, and we propose the following solutions to build upon these efforts and combat barriers unique to applicants without HRPs 23 24 ( Table 1 ).

**Table 1:** Proposed solutions to better support applicants without home residency programs

Theme	Supporting findings	Recommendation
Mentorship is a critical component to Match success	Applicant without HRPs were more likely to match if they had identified mentors in plastic surgery	Create formalized mentorship programs with already established ACAPS sister institutions, based on proven strategies in other surgical subspecialties
Peers represent alternate sources of mentorship	Outside the attendings, applicants cited peer mentorship as their most utilized resource when applying into plastic surgery	Investigate the current prevalence of plastic surgery interest groups and form additional groups associated with national organizations
Applicants are turning toward social media to inform their Match process	Over half of matched applicants reporting using plastic surgery social media during their Match process	Encourage the establishments of social media presence by all plastic surgery residency programs
Applicants without access to a plastic surgery division are at an increased disadvantage in the Match	Disparities found in applicants without HRPs were exacerbated in those who also did not have access to a plastic surgery division	Establish national or regional grants targeted toward students without HRPs to bolster their access to research opportunities

Abbreviations: ACAPS, American Council of Academic Plastic Surgeons; HRP, home residency program.

### Improving Mentorship Pairing

Medical student mentorship has long been an area of interest, and the positive effects of having a mentor have been clearly demonstrated. 25 26 This is no exception for students without HRPs as our data showed that students from HRPs who identified a mentor in PS were more likely to match. Successful strategies for establishing mentorship included cold-calling/emailing and performing research; however, these methods seem to place a large amount of onus on the student to establish a relationship. A study performed by Sasson et al demonstrated that nearly 60% of students interested in PS reported difficulties establishing a mentor–mentee relationship because they simply did not know where to find one. 11 The American Council of Academic Plastic Surgeons (ACAPS) has since developed a database to pair institutions without HRPs with sister institutions with residency programs. 27 This database consists of name and contact information of sister institutions' program directors for 103 medical schools without HRPs. 11 Additionally, a formalized mentorship program between these sister institutions has been trialed by ACAPS, with over 30 students receiving mentors. A similar program has been shown to be successful in the field of urology, where 94% of participating students successfully matched. 28

### Peer Mentorship

In addition to mentorship from attendings and residents, our study highlights the importance of peer mentorship with 74% of matched students having reported utilizing help from senior students. Within medical schools, student interest groups represent an easily accessible and approachable way for students to develop peer mentorship, gain exposure to the field, and discover career-building opportunities. In other specialties such as orthopaedic surgery, it has been shown that a majority of U.S. allopathic schools have a student interest group. 29 However, similar work is yet to be done within PS, especially examining the presence of interest groups at institutions without HRPs. 29 Moving forward, we believe that it is important to characterize the prevalence of PS interest groups. Additionally, we propose forming a partnership with national societies such as ACAPS or the American Society of Plastic Surgeons to create official chapters at medical schools without HRPs. A similar concept has been done with the American Association of Neurological Surgeons with more active chapters being associated with increased residency match success. 30

### Social Media as a Tool for Education

Social media has become a powerful tool for plastic surgeons and PS programs to brand themselves and disseminate information. A study performed in 2020 revealed that over 85% of integrated PS residency programs had Instagram accounts with 44% of posts pertaining to either education, promotion of PS, or resident life. 31 These resources are openly available to applicants without HRPs and massively expand their ability to connect and learn, when previously their interest in PS would be limited by institutional access and geographic base. 31 Our data show that applicants without HRPs are already taking advantage of these resources with 52% of matched applicants using Twitter, Instagram, or Facebook as a resource for PS. Since the COVID-19 pandemic, there has been an overall increase in social media presence by U.S. residency programs. Social media has become a powerful tool for plastic surgeons and PS programs to brand themselves and disseminate information. A 2018 study performed by Chandawarkar et al demonstrated that only 21% of integrated U.S. PS residency programs had active Instagram accounts. 32 While this statistic is outdated, the point stands that all PS residency programs should strive to maintain some form of social media presence. 32 As this appears to be a lasting trend, we encourage applicants without HRPs to continue to access residency program social media pages as a resource during their Match process.

### The Value of Surgical Divisions

In their article titled “Building an Academic Colorectal Division,” Koltun et al describe the efforts required to create a formal surgical division. 33 He lays out critical factors to the formation of a division, which includes culture, commitment, collaboration, control, cost, and compensation. 33 Within these factors, Koltun et al also highlight the importance of developing a system of mentorship and focused research. Similar studies have been performed within PS, which address the eventual creation of departments from divisions. 34 Our data demonstrate that a significant barrier for PS applicants without an HRP or home division is difficulty in identifying research opportunities. It is possible that the organizational infrastructure associated with surgical divisions aids applicants in obtaining research opportunities and may later improve their outcomes in the Match. However, the creation of an academic surgical division is a timely process, requiring manpower, time, and financial resources. As a substitute, we encourage the establishment of national or regional grants targeted toward students without HRPs. A similar effort has already been done for minority students.

In conclusion, the results of this study emphasize the importance of obtaining a PS mentor for students without HRPs. This study also revealed that applicants who lack both an HRP and home division face additional barriers to matching. We believe that this population is further disadvantaged due to their lack of exposure to the field and limited access to research opportunities and mentorship. When assessing a PS applicant, program directors should be aware of the additional barriers that these candidates face. These learning points can be applied to the upcoming application cycle to improve the overall experience and results for programs and medical students alike.

There are several limitations to our study. Although there is a representation of applicants and residents from various institutions, the overall response rate was low. We were unable to capture all PS applicants, particularly those who participated in the supplemental offer and acceptance program or who ultimately matched into general surgery. Additionally, as a survey-based study, our results are subject to response bias, possibly selecting applicants who were successful in their Match and impacting our match rate data. Recall bias is also of concern for PGY5 resident responses as they are commenting on their perceptions from over 5 years prior. In addition, there is also subjectivity in the interpretation of some questions that may lead to variability in survey responses.
